# Graphene supremacy: the duo duo

**DOI:** 10.1093/nsr/nwac233

**Published:** 2022-10-22

**Authors:** Fan Zhang, Xin-Cheng Xie

**Affiliations:** Department of Physics, University of Texas at Dallas, USA; International Center for Quantum Materials, School of Physics, Peking University, China

Graphene is an amazing material that presents us with new challenges and opportunities, even after two decades of extensive study. The two linear energy bands of graphene cross at the *K* and *K’* points of the hexagonal Brillouin zone with topological winding numbers ±1. This Dirac cone description is nearly perfect within a ∼3 eV energy range—more than 100 times room temperature. Moreover, the chemistry of graphene sets its Fermi energy right at the Dirac points. A historic question, first posted by Linus Pauling, is whether the Dirac cone can be gapped as a result of the electron–electron interactions [1]. The affirmative is a solid-state analog to the spontaneous chiral symmetry breaking in the Standard Model of elementary particles. However, graphene does not directly render strongly interacting phases—the holy grail of quantum condensed matter physics—in the absence of a strong magnetic field [2] because its bare electron–electron interaction is not sufficiently strong and because its Dirac velocity characterizing its kinetic energy is not sufficiently small.

About a decade ago, two graphene architectures were theoretically shown to produce strongly interacting phases. One is an artificial architecture formed by overlaying two graphene layers with a small twist angle [3]. The other is a natural architecture formed by few-layer graphene with a rhombohedral stacking order [4]. Now, in a landmark experiment, the team led by Feng Miao at Nanjing University has ingeniously synthesized the natural and artificial architectures and, for the first time, provided compelling evidence for realizing quantum pseudo and two-stage criticalities and their *in situ* manipulation via electric and magnetic fields [5].

For twisted bilayer graphene, the Dirac velocity drops to zero as the twist angle turns from 30° to ∼1°, dubbed the magic angle [3]. Consequently, superconductivity, ferromagnetism, nematicity and the quantum anomalous Hall effect have all been experimentally observed in the spontaneous symmetry breaking near the magic angle [6]. A prime example of the rhombohedral graphene is the AB-stacked bilayer. Such an *N*-layer system generalizes the monolayer's features: a pair of bands of approximately ±*p^N^* dispersions touch at the *K* and *K’* points with the topological winding number ±*N* [4]. At charge neutrality, a family of spontaneously gapped, valley-projected topological phases have been identified [4,7,8]. At extremely light doping, trigonal warping dramatically distorts the flat bands, yielding van Hove singularities; Stoner phases, Wigner-Hall crystals, strange metals and unconventional superconductors have been observed recently [9,10].

**Figure 1.**
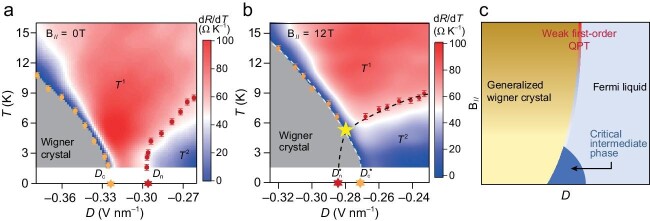
(a and b) Phase diagrams of (*π* + 0.75°) twisted double bilayer graphene at the in-plane magnetic *B* = 0 and 12 T, respectively. (c) Schematic of the phase diagram at 0 K. Adapted from ref. [5].

The magic occurs when two AB bilayer graphene, the duo duo, are twisted by 0.75° over *π* and the second moiré band is filled in a way such that every three moiré unit cells lack one electron [5]. At large out-of-plane electric fields, the electrons ‘crystallize’ themselves and the system behaves like an insulating Wigner crystal, as revealed by its Efros–Shklovskii scaling behavior of the temperature dependence of resistance. At small fields, the system is restored to a normal metal with quadratic temperature dependence. At intermediate fields, a strange metal emerges, as the resistance exhibits linear temperature dependence close to the Planckian dissipation limit. As shown in Fig. 1a, the electric field-driven quantum melting of the Wigner crystal phase undergoes a two-stage transition. When an in-plane magnetic field is applied, the critical strange metal regime shrinks, as sketched in Fig. 1c, and eventually only exists above a critical temperature, as shown in Fig. 1b. Meanwhile, the universal scaling behavior in the other two regimes do not persist below the same critical temperature, indicating the emergence of a quantum pseudo criticality.

These significant results [5] call for future works to further substantiate the realization of an extended Hubbard model using twisted double bilayer graphene and to enrich the understanding of the rare phases observed in it, particularly from more direct microscopy and spectroscopy studies [6]. It is a mystery why the first moiré band or other commensurate fillings do not yield strongly interacting phases. One may also wonder how robust the discovered phases and their transitions are against the twist angle and its inevitable inhomogeneity [6]. However, there is no doubt that the work by Feng Miao's team has established a well-grounded versatile quantum simulator for exploring and manipulating strongly correlated electron physics, by taking the advantage of the superb customizability of designing 2D materials stacks. The existence of many more convenient tuning knobs and a greater number of unit cells will likely turn out to be another superb advantage over more established techniques that require exhaustive computation or precise control of cold atoms. Graphene-based quantum simulation has been turned on by the first generation of pioneering works including this highlighted one [5]. Stay tuned.

***Conflict of interest statement.*** None declared.
